# Mid-term outcomes of hypogastric artery embolization in endovascular aneurysm repair: a case series

**DOI:** 10.1093/jscr/rjae029

**Published:** 2024-02-06

**Authors:** Takeyuki Kanemura, Yoshinori Nakahara, Retsu Tateishi, Fumiya Haba, Shunya Ono

**Affiliations:** Department of Cardiovascular Surgery, IMS Katsushika Heart Center, 3-30-1 Horikiri, Katsushika Ward, Tokyo 124-0006, Japan; Department of Cardiovascular Surgery, IMS Katsushika Heart Center, 3-30-1 Horikiri, Katsushika Ward, Tokyo 124-0006, Japan; Department of Cardiovascular Surgery, IMS Katsushika Heart Center, 3-30-1 Horikiri, Katsushika Ward, Tokyo 124-0006, Japan; Department of Cardiovascular Surgery, IMS Katsushika Heart Center, 3-30-1 Horikiri, Katsushika Ward, Tokyo 124-0006, Japan; Department of Cardiovascular Surgery, IMS Katsushika Heart Center, 3-30-1 Horikiri, Katsushika Ward, Tokyo 124-0006, Japan

**Keywords:** abdominal aortic aneurysm, endoleak, endovascular aneurysm repair, hypogastric artery embolization

## Abstract

Hypogastric artery embolization is performed during endovascular aneurysm repair (EVAR) involving the common iliac artery. Within this case series, we have observed elevated rates of sac expansion subsequent to this intervention. April 2009 to March 2021, 22 patients underwent EVAR with hypogastric artery embolization. We evaluated the mid-term outcomes for these patients. The mean follow-up period was 57 months. We achieved a 100% technical success rate without open conversion and no hospital deaths. The rates of freedom from aneurysm expansion at 1, 3, and 5 years were 90.5%, 59.1%, and 37.5%, respectively. The percentage of sac expansion exceeding 5 mm was 54.5% (12/22). Combined endovascular aortic aneurysm repair and embolization of the hypogastric artery might be associated with a high rate of remote sac expansion. Larger trials are needed to verify risks and benefits.

## Introduction

Hypogastric artery embolization is not routinely performed during endovascular aneurysm repair (EVAR) procedures, but it is necessary in 20–32% of cases involving the common iliac artery (CIA) [1–5]. When the distal landing zone in the CIA is too short to anchor, extending the endograft limb into the external iliac artery is required. Thus, hypogastric artery embolization is necessary to prevent Type II endoleaks. However, concerns have arisen regarding the safety of hypogastric artery embolization due to reports of heightened buttock claudication and erectile dysfunction [6], along with increased occurrences of ischemic colitis and prolonged hospital stays [7]. Furthermore, long-term results are infrequently documented. We hypothesized that embolization of the hypogastric artery may lead to the formation of medial pelvic collateral vessels, potentially increasing the risk of Type II endoleaks and subsequent sac expansion. To test this hypothesis, we evaluated the case series who underwent simultaneous embolization and EVAR.

## Case series

In this case series, 22 (5.2%) patients who underwent concomitant hypogastric artery embolization were selected for further analysis. We evaluated the overall survival and freedom from aneurysm expansion rates, and the early and late complications, including endoleaks and absence of aneurysm expansion. All patients underwent preoperative imaging using contrast-enhanced computed tomography (CT) scans. All EVAR procedures were performed in the operating room with the aid of a C-arm under general anesthesia via a cut-down approach to the femoral artery. The devices used for the procedures were selected according to the surgeon’s preference. Embolization of the hypogastric artery was performed in all patients, with coils used in 20 patients and AMPLATZER Vascular Plug 4 (St. Jude Medical, Inc, St. Paul, Minnesota, USA) in two patients. The patients were followed up postoperatively with CT at 3, 6, 12, 18, and 24 months and annually thereafter. Except in one patient who underwent a non-contrast CT scan because of renal dysfunction, all patients underwent contrast-enhanced CT scans.

Primary technical success was defined as successful introduction and deployment of the device and was evaluated on an intent-to-treat basis, following the reporting standards established by The Society for Vascular Surgery and The American Association for Vascular Surgery [8]. Overall survival, rupture-free survival, and absence of aneurysm expansion were analyzed using the Kaplan–Meier method. Early surgical outcomes and late complications were also analyzed. Continuous data were presented as the median and interquartile range (25th–75th percentile) or as the mean and standard deviation, as appropriate. All statistical analyses were performed using R 3.2.3 software and EZR (Saitama Medical Center, Jichi Medical University, Saitama, Japan), a graphical user interface for R [9].

## Results

### Patient characteristics

The mean patient age was 73.6 ± 16.0 years, and 18 of the patients were male. The patient characteristics are summarized in Table 1. The mean creatinine level was 1.28 ± 1.4 mg/dL; mean aneurysm diameter, 46.9 ± 12.3 mm; mean neck length, 37 ± 17 mm; and mean neck diameter, 20.3 ± 3.2 mm. In 11 patients, the aortic tortuosity was Grade III (<120°).

**Table 1:** Clinicodemographic patient characteristics.

**Demographics**	**Overall**
Age, years	73.6 ± 16.0
Male sex (%)	18 (81.8)
Smoking (%)	15 (68.2)
Hypertension (%)	20 (90.9)
Diabetes mellitus (%)	4 (18.2)
Dyslipidemia (%)	8 (36.4)
Chronic kidney disease (%)	6 (27.3)
Creatinine, mg/dl	1.28 ± 1.14
COPD (%)	2 (9.1)
Coronary artery disease (%)	8 (36.4)
Antiplatelet agent (%)	9 (40.9)
Anticoagulation agent (%)	4 (18.2)
Primary EVAR (%)	20 (87.0)
Hostile neck (%)	22 (100.0)
Tortuosity of the aorta	
Grade I: 150–180°	5 (22.7%)
Grade II: 120–150°	5 (22.7%)
Grade III: <120°	11 (47.8%)
Neck diameter, mm	20.3 ± 3.1
Neck length, mm	37 ± 17
Maximum of sac diameter, mm	46.9 ± 12.3
Patent IMA (%)	13 (59%)
IMA diameter, mm	2.5 ± 0.7
Maximum diameter of lumbar artery, mm	1.8 ± 0.6
Numbers of patent lumbar artery	
≤4	12 (54.5%)
>4, ≤6	8 (36.4%)
>6	1 (4.5%)

### Early outcomes

Prior to EVAR, all patients underwent embolization of one or both sides of the hypogastric artery. Bilateral embolization was executed in two stages, with a minimum interval of 1 week between each phase. As shown in Table 2, the most common cause of hypogastric artery embolization was a short distal landing length (*n* = 12 patients, 54.5%), followed by CIA aneurysms (*n* = 5 patients, 22.7%), and hypogastric artery aneurysms (*n* = 8 patients, 36.4%). A bifurcated device was utilized in 20 patients, and a single-leg device was utilized in two patients. The additional procedures are listed in Table 2. The procedures were declared as primary successes, without the requirement for conversion to open surgery.

**Table 2:** Intraoperative variables and early postoperative complication.

**Intraoperative variables**	
Operation time, mins	196 ± 70
Fluoroscopy time, mins	62 ± 26
Iodine contrast medium, ml	93 ± 58
Primary success (%)	22 (100)
Device	
Zenith (%)	7 (31.8)
EXCLUDER (%)	7 (31.8)
ENDURANT (%)	7 (31.8)
Talent (%)	1 (4.5)
Cause of internal iliac artery embolization (%)	
Short distal landing length	12 (54.5)
common iliac artery aneurysm	5 (22.7)
internal iliac artery aneurysm	8 (36.4)
Additional procedure (%)	
Bilateral iliac artery embolization	2 (9.1)
Iliac angioplasty	3 (13.6)
Femoro-femoral crossover bypass	1 (4.5)
Internal iliac artery embolization	22 (100.0)
Internal iliac artery-external iliac artery bypass	3 (13.6)
Early postoperative complication	
Cardiac	0
Pulmonary	0
Urinary tract infection	0
Sepsis	0
Ischemic colitis	0
Peripheral embolization	0
Buttock claudication (%)	2 (9.1)
Access site bleeding or hematoma	0
Lymph leakage (%)	1 (4.5)
Dissection of aorta (%)	3 (13.6)
30-Day mortality	0

The mean operative time was 196 ± 70 min; mean fluoroscopy time, 62 ± 26 min; and mean amount of iodine contrast medium used, 93 ± 58 mL. The procedural success rate was 100%. Four different devices were used: ZENITH (Cook Inc, Bloomington, Indiana, USA) (*n* = 7 patients, 31.8%), EXCLUDER (W.L.GORE & Associates, Flagstaff, Arizona, USA) (*n* = 7 patients, 31.8%), ENDURANT (Medtronic, Minneapolis, Minnesota, USA) (*n* = 7 patients, 31.8%), and TALENT (Medtronic, Minneapolis, Minnesota, USA) (*n* = 1 patient, 4.5%). The postoperative complications are summarized in Table 2. There were no cases of cardiac, pulmonary, or urinary tract infection; sepsis; ischemic colitis; peripheral embolization; or access site bleeding/hematoma. However, two cases (9.1%) of buttock claudication and one case (4.5%) of lymph leakage were reported, along with three cases (13.6%) of Type B aortic dissection. No cases of 30-day mortality were reported.

### Late outcomes

**Figure 1:**
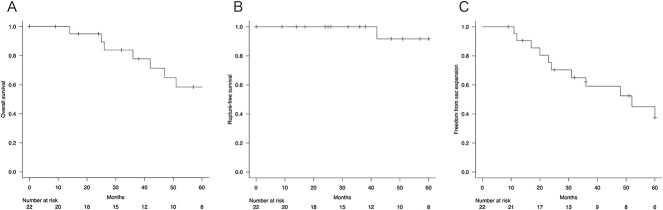
(A) Overall survival. (B) Rupture-free survival. (C) Freedom from sac expansion.

The estimated overall survival rate was 100% at 1 year, 77.8% ± 9.8% at 3 years, and 58.4% ± 12.4% at 5 years (Fig. 1A). Two patients died of rupture of the aneurysm during the follow-up period. The rupture-free survival was 100% at 1 year, 100% at 3 years, and 91.7% ± 8.0% at 5 years (Fig. 1B). The mean aneurysm sac diameter at 3 and 5 years postoperatively was 51.8 ± 15.9 mm and 59.8 ± 15.8 mm, respectively. Shrinkage of the aneurysm sac (≥5 mm) was observed in 2 of 22 patients (9.1%), with a freedom from aneurysm expansion ratio of 95.2% ± 4.7% at 1 year, 59.1% ± 11.3% at 3 years, and 37.5% ± 12.3% at 5 years (Fig. 1C). Late complications were observed in 14 patients, including ruptures, reinterventions, sac expansion, and secondary endoleaks in 2, 4, 12, and 4 patients, respectively. Type I endoleaks requiring reintervention occurred in two patients, but no Type III endoleaks or migration occurred. The incidence of endoleaks is shown in Table 3. Despite sac expansion, four patients declined reintervention because of advanced age or associated complications, so they were only monitored.

**Table 3:** Occurrences of endoleaks, late aneurysm ruptures, and late conversion to open surgery throughout the follow-up period.

**Event**	<6 months	6 months–1 year	1–3 years	3–5 years	>5 years	Total events (n)
Type Ia Endoleak	1					1
Type Ib Endoleak						0
Type II Endoleak	5	4				9
Late aneurysm rupture				1	1	2
Late conversion to open surgery			1			1

**Figure 2:**
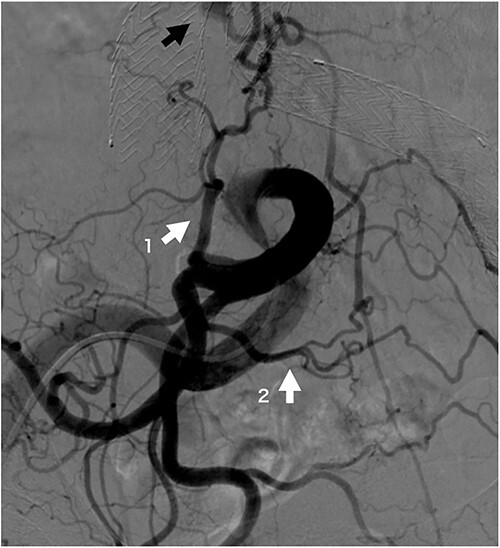
Iliac artery angiogram on opposite side of coil embolization procedure site at 6 months post-operation. The iliolumbar artery (white arrow 1), a branch of the internal iliac artery, and the lateral sacral artery (white arrow 2), a branch of the superior gluteal artery, are well developed and form a rich arcade. The black arrow indicates the sac refilling flow.

Four patients required reinterventions during follow-up, with one patient presenting with a Type Ia endoleak and undergoing open surgery at 33 months postoperatively, which involved banding of the proximal neck. Another patient was diagnosed with a Type II endoleak from the lumbar artery and underwent coil embolization 6 months postoperatively. After performing an iliac artery angiogram, abundant collateral flow, which included the iliolumbar artery leading to the lumbar artery and subsequently to the sac, was observed (Fig. 2). The third patient underwent distal endograft extension for a Type Ib endoleak at 24 months postoperatively. The last patient underwent aortic angiography and was diagnosed with a Type II endoleak but declined further surgical treatment because of advanced age.

## Discussion

The guidelines from the Society for Vascular Surgery state that the hypogastric artery can be unilaterally embolized with minimal adverse events during EVAR procedures [10]. However, the long-term results remain unclear. This study found a high incidence of persistent Type II endoleak and a significant proportion of patients exhibiting sac expansion. The frequency of persistent Type II endoleaks was alarmingly high at 40.9% (9/22).

The risk factors for Type II endoleak after EVAR include distal graft extension, older age, absence of chronic obstructive pulmonary disease (COPD), patent inferior mesenteric artery (IMA), and increased number of patent lumbar arteries [11–13]. However, the relationship between embolization of the hypogastric artery and incidence of Type II endoleaks after EVAR for abdominal aortic aneurysms remains unclear. We speculate that this phenomenon is attributable to the proliferation of collateral circulation within the pelvic region, consequent to the embolization of the hypogastric artery, which leads to Type II endoleaks. The pelvic region has abundant collateral circulatory pathways, and the alteration of pelvic circulation following embolization of the hypogastric artery is well documented [14]. Particularly, the hypogastric, median sacral, and lumbar arteries form a closely interrelated arcade, and it is expected that the collateral vessels will have increased development after embolization of the hypogastric artery, leading to Type II endoleaks. Recent reports have revealed that Type II endoleaks occurred in 20–30% of typical EVAR procedures [15–17], and this is significantly lower than the incidence in the present study. Literature detailing the incidence of Type II endoleaks after hypogastric artery embolization is scarce, and thus, future studies are eagerly awaited.

In our case of reintervention for Type II endoleaks, the hypogastric artery on the opposite side of the embolization demonstrated abundant blood flow to the median sacral and lumbar arteries. This phenomenon can be attributed to the hypogastric artery route, which involves the contralateral hypogastric artery, lateral sacral artery, and ipsilateral hypogastric artery [14]. An increase in blood flow in the superior gluteal artery, located upstream of the lateral sacral artery, results in augmented flow to its branch (i.e. the median sacral artery) that then drains into the sac. This increased blood flow is a Type II endoleak, validating our hypothesis.

A previous report found that compared with patients with non-CIA aneurysm, those with concomitant CIA aneurysm experienced a higher rate of reintervention after EVAR for abdominal aortic aneurysm [18], and consistent findings were found in the present study. Additionally, the EUROSTAR study showed that concomitant iliac artery aneurysms in patients with abdominal aortic aneurysms were associated with an increased incidence of distal Type I endoleaks, hypogastric aneurysms, and aneurysm rupture [19]. These reports do not explore the specifics of Type II endoleaks; however, it is plausible that the Type II endoleaks may have contributed to aneurysm enlargement and subsequent endoleak Type I and reintervention, as observed in the current study.

In the mid-term analysis, 54.5% (12/22) of patients had sac expansion. In a large series, the rate of aortic sac expansion after EVAR was 21% after 5 years [20]. Another study involving 157 EVAR patients reported a rate of 25% at 3 years [21]. Compared to these reports, the current study found that 54.5% of the patients had a poor outcome. In another study, among patients treated for Type II endoleaks based on surveillance-detected abdominal aortic aneurysm sac expansion, 55% continued to show expansion ˃5 mm 5 years after treatment [22]. Our findings align with this result. Sac expansion is possibly caused by the high frequency of Type II endoleaks following EVAR with hypogastric artery embolization.

The limitations of the present study include its retrospective and observational design and the small sample size. Additionally, the small number of coil embolizations for the IMA and lumbar artery may also have influenced the occurrence of Type II endoleaks. The findings need to be validated in studies with a larger sample size. Larger patient population trials are also needed to verify the risks and benefits of concomitant embolization of the hypogastric artery.

In conclusion, a combination of EVAR and hypogastric artery embolization might be associated with an incidence of remote sac expansion.
